# Identification of all-against-all protein–protein interactions based on deep hash learning

**DOI:** 10.1186/s12859-022-04811-x

**Published:** 2022-07-08

**Authors:** Yue Jiang, Yuxuan Wang, Lin Shen, Donald A. Adjeroh, Zhidong Liu, Jie Lin

**Affiliations:** 1 College of Computer and Cyber Security, Fujian Normal University, Fuzhou, 350108 People’s Republic of China; 2 No. 2 Thoracic Surgery Department Beijing Chest Hospital, Capital Medical University, Beijing Tuberculosis and Thoracic Tumor Research Institute, Beijing, 101149 People’s Republic of China; 3 Lane Department of Computer Science and Electrical Engineering, West Virginia University, Morgantown, 26506 USA

**Keywords:** Protein–protein interaction, Deep learning, Binary hash code, Binary search, Hamming distance

## Abstract

**Background:**

Protein–protein interaction (PPI) is vital for life processes, disease treatment, and drug discovery. The computational prediction of PPI is relatively inexpensive and efficient when compared to traditional wet-lab experiments. Given a new protein, one may wish to find whether the protein has any PPI relationship with other existing proteins. Current computational PPI prediction methods usually compare the new protein to existing proteins one by one in a pairwise manner. This is time consuming.

**Results:**

In this work, we propose a more efficient model, called deep hash learning protein-and-protein interaction (DHL-PPI), to predict all-against-all PPI relationships in a database of proteins. First, DHL-PPI encodes a protein sequence into a binary hash code based on deep features extracted from the protein sequences using deep learning techniques. This encoding scheme enables us to turn the PPI discrimination problem into a much simpler searching problem. The binary hash code for a protein sequence can be regarded as a number. Thus, in the pre-screening stage of DHL-PPI, the string matching problem of comparing a protein sequence against a database with *M* proteins can be transformed into a much more simpler problem: to find a number inside a sorted array of length *M*. This pre-screening process narrows down the search to a much smaller set of candidate proteins for further confirmation. As a final step, DHL-PPI uses the Hamming distance to verify the final PPI relationship.

**Conclusions:**

The experimental results confirmed that DHL-PPI is feasible and effective. Using a dataset with strictly negative PPI examples of four species, DHL-PPI is shown to be superior or competitive when compared to the other state-of-the-art methods in terms of precision, recall or F1 score. Furthermore, in the prediction stage, the proposed DHL-PPI reduced the time complexity from $O(M^2)$ to $O(M\log M)$ for performing an all-against-all PPI prediction for a database with *M* proteins. With the proposed approach, a protein database can be preprocessed and stored for later search using the proposed encoding scheme. This can provide a more efficient way to cope with the rapidly increasing volume of protein datasets.

## Background

Protein–Protein Interaction (PPI) in biological cells is vital for molecular processes and biochemical reactions, such as intracellular communications, signal transduction and gene regulation. Hence,the identification of PPI is important in life process research, disease diagnosis and treatment, and in drug development [1–3].

To identify PPI using wet-lab experiments is costly and time consuming. Though current high throughput methods have significantly improved the efficiency and cost, for instance using yeast 2-hybrid (Y2H) [4], mass spectrometric protein complex identification (MS-PCI) [5], Co-Immunoprecipitation (Co-IP) [6], and Tandem affinity purification-mass spectrometry (TAP-MS) [7], the wet lab methods are still expensive and often results in many false positives and false negatives  [8]. Computational identification methods are often used to pre-screen and predict PPIs before the wet-lab experiments, given the convenience, efficiency, and improving effectiveness of algorithmic approaches.

The protein sequence is a key element in algorithmic approaches to PPI prediction. The existing sequence-based PPI recognition methods can be divided into three basic groups: co-occurrence based, pattern matching based, and machine learning based methods.

The methods based on co-occurrence [9] judge the potential interaction between a pair of proteins by counting the frequency of their co-occurring patterns. Bunescu et al. [10] recognized the protein–protein interaction by extracting frequent patterns. One problem with use of frequent patterns and co-occurrence approaches is that they generally achieve high recall but low precision and poor generalization [11].

Pattern matching based methods search for potential PPI by establishing certain pattern rules. Fundal [12] proposed the dependency relationship based on the structure of syntax rules of sentences. Temkin [13] distinguished PPI through the sentence analyzer with the rules of grammar generation. This method needs to construct patterns manually, thus it is inefficient, time-consuming and labor-intensive [14]. Moreover, because of the diversity of relational patterns of PPI, the predefined rules may not always cover all PPI rational patterns. Both pattern matching and co-occurrence based methods often involve the use of efficient search data structures such as suffix trees and suffix arrays [15] for improved processing time.

Kernel based methods are often used to reduce the problems of predefining pattern rules. Haussler et al. [16] presented the convolution kernel which can be used on discrete structures. The string kernel was proposed by Lodhi et al. [17], and it used the inner product of the word substring with a specific length in the feature space. Kernel-based approaches analyze PPI according to the grammar, syntax and dependency of a single sentence. However, due to the complex grammar and the possibly indirect manifestation of PPI, the resulting predictions may not have high accuracy. The kernel-based approaches can exploit various properties of proteins as features, for instance, physicochemical properties such as hydrophobicity profiles, amino acid composition, and domain composition, genomic features such as gene neighbourhoods, and features based on network topology [18].

The machine learning based PPI analysis methods can be classified into supervised or unsupervised, depending on whether they used labeled data, or unlabeled data. The supervised methods for PPI prediction learn the relevant mapping functions from labeled PPI data, and then predicts PPIs. Frequently used supervised machine learning methods include decision trees [19, 20], support vector machines (SVM)  [21–24], artificial neural networks (ANN)  [25–27], *k*-nearest neighbors (KNN) [28] and Naive Bayes [29]. Unsupervised PPI analysis methods learn the intrinsic feature representation of the unlabeled data, and then carry on deeper analysis. For instance, the method of *k*-means is often applied on PPI clustering problems [30, 31]. Some related methods on predicting protein-ncRNA interactions (for instance, [32, 33]) have used a combination of pattern matching and machine learning methods.

More recently, machine learning methods based on deep learning[34], has been widely used for PPI prediction, with remarkable results. Zhao et al. [35] used nine properties of amino acids as feature representation (including Relative Exterior Solvent Accessible area (RESA) and Hydropathy Index (HI)), and then trained Long-Short Term Memory (LSTM) [36] networks to predict interface residue pairs from two monomer proteins. Li et al. [37] firstly substituted corresponding random numbers for amino acids in the protein sequence to complete sequence coding. Then they mapped amino acids to dense vector by using *word*2*vec* through an embedding layer. Next, they exploited the potential long term dependence between amino acids by using Convolution Neural Networks (CNN) [34] and LSTM. Finally, the learnt features are fed into the fully connected layer to predict PPIs. Somaye et al. [38] divided a protein into subsequences, and used these subsequences to perform multiple sequence alignment through PSI-BLAST to obtain a protein profile. This is then fed to a convolutional module and a random projection module to predict PPIs. Sun [39] combined autocovariance(AC) and conjoint triad(CT) as feature representation for the proteins, and used these to perform PPI prediction between protein sequences using stacked a autoencoder (SAE) [34], where AC used seven physicochemical properties of amino acids and CT described the composition of amino acids. Similarly, Du et al. [40] analyzed the composition of amino acids using three considerations – composition, transition and distribution, while using quasi-sequence-order descriptors, Amphiphilic Pseudoamino Acid Composition(APAAC) to represent the physical and chemical properties of the proteins. Then they learned protein representations using two different deep neural (DNN) network approaches. Sunil [41] presented a protein domain-based method, DeepInteract, which taking the protein domain frequency as a feature. It used 2,971 protein domains and performed PPI prediction using DNN.

Graph based deep learning methods construct networks according to proteins and their relationships, typically using the proteins as nodes, and the interactions between them as edges. Huang [42] used the adjacency matrix to represent the interaction between proteins in a graph. They simulated the evolution process of the PPI network implicitly using SAE, and finally predicted PPIs using a regularized Laplacian kernel. One problem with this approach is the significant sparseness of the network, since most of the proteins would not be interacting. Fang et al. [43] attempted to reduce the problem of sparseness by representing the protein sequence by using the conjoint triad (CT), and further learned the embedding of protein sequences using local information extracted from the sequences via a signed variational graph autoencoder (S-VGAE).

A major problem in analyzing PPIs is the huge number of proteins in a natural environment. This makes it difficult and too expensive to verify all potential PPIs between every pair of proteins. This is the all-against-all PPI problem. This difficulty typically leads to the problem of incompleteness in current known PPI networks [44]. Given a dataset with *M* protein sequences, in order to predict the potential PPIs among them, current approaches compare each protein to every other protein one by one in a pairwise manner. This will require comparison for $\frac{M(M-1)}{2}$ protein pairs, that is, with $O(M^2)$ time complexity. In this work, we propose DHL-PPI that solves the all-against-all PPI problem in a reduced time complexity of $O(M \log _{2} M)$.

The proposed DHL-PPI method transforms a protein sequence into a binarized hash code using deep learning techniques. In the pre-screening stage, DHL-PPI takes part of a binarized hash query code which can be regarded as a number, to search against the same part of binarized hash ontic codes in a database with *M* proteins, which can be regarded as an array with *M* numbers inside. Then the string matching problem is turned into a problem of finding exact number inside an array. After this pre-screening stage, searching on the whole database can be narrowed down to a much smaller candidate set. This smaller candidate set is further processed by calculating the Hamming distance between the query hash code and ontic hash codes to determine whether there is PPI relationship or not. On average, the proposed DHL-PPI only needs $O(M \log _{2} M )$ time complexity to predict all PPI in a database with *M* proteins. This reduces the time complexity and improves the search performance significantly.

The experimental results confirm that DHL-PPI is feasible and effective for PPI prediction. On a dataset with strictly negative PPI examples of four species data, DHL-PPI  is superior to, or competitive with, the other methods in terms of precision, recall, and F1 score. The results demonstrate that the proposed DHL-PPI is suitable to predict PPI accurately in much simpler, faster, and efficient way. For a given dataset, the hash codes can be computed for each protein and stored for later search.

The remainder of the paper is organized as follows. In “Experiments and results” section, we show our experiments and results, including a brief description of the datasets, evaluation criteria, and results. In “Discussion” section, we discuss the experimental results. In “Conclusion” section, we draw our conclusions. In “Methods” section, we present details of the methods, including the algorithms used and their complexity analysis.

## Experiments and results

The experimental dataset contains information on protein sequences, including both pairs with known PPI, and those known to have no PPI. In this benchmark data set, we verify the validation of the proposed DHL-PPI method. For the experiments, we adopted the standard 10-way cross validation (CV) approach.

### Dataset

The dataset is from the Human Protein References Database (HPRD) [45] (http://www.hprd.org/, release 7_20070901). Pan et al. [46] used this dataset for PPI prediction, where all positive PPI relationships and negative PPI relationships are confirmed using wet lab experiments.

The dataset further contains information on four species [46], namely, (1) *Caenorhabditis elegans* (nematode worm) dataset; (2) *Drosophila melanogaster* (fruit fly) dataset; (3) *Escherichia coli* dataset; and (4) *Homo sapiens* (Human) dataset. The negative set of the dataset is generated based on the hypothesis that two proteins in different cellular compartments do not have any interaction. More details on the criteria are described in Yang [43]. Therefore, the negative set of the dataset [46] is regarded as “strictly negative samples” in the field. The number of proteins, positive samples, and negative samples for each of the four species in the dataset are shown in Table 1.

**Table 1:** Description of the dataset on the four species used in our experiments

Dataset	Number of proteins	Number of positive samples	Number of negative samples
*C. elegans* dataset	1734	2877	1670
Drosophila dataset	5624	19712	14900
*E. coli* dataset	1528	5576	4031
Human dataset	7803	31761	25203

**Fig. 1:**
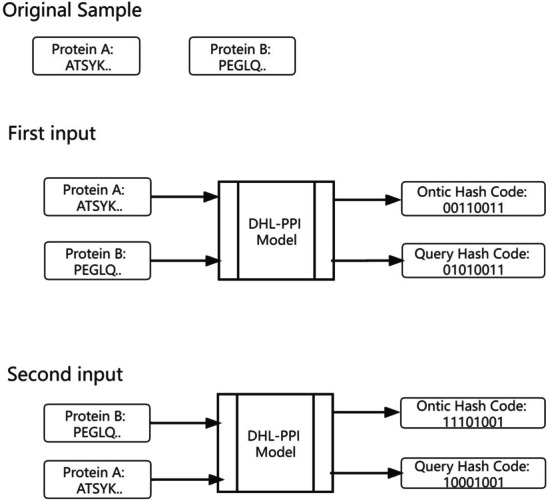
An example for input a pair of protein A and protein B

In this work, we used 10-way cross-validation. The data with the positive samples and negative samples are randomly divided into training set and testing set with ratio of 9 : 1. The adopted measurements are the average of the test runs from the 10-way cross validation. For an input protein pair, A and B, when A is used to generate an ontic code, B will be used to generate a query code. And then, the roles are reversed in the next input, protein A is used to generate query code and protein B is used to generate an ontic code. An example is shown in Fig. 1. Thus, the used samples are doubled in all four species.

### Evaluation criteria

In this work, we use measurements derived from confusion matrix to evaluate our models. These measurements are precision, recall, *F*1, specificity, accuracy, and Mathews correlation coefficient (MCC). Their definitions are presented as follows respectively:

$$\begin{aligned}&precision=\frac{TP}{TP+FP} \\&recall=\frac{TP}{TP+FN} \\&F1=\frac{2\times precision \times recall}{precision+recall} \\&Specificity=\frac{TN}{TN+FP} \\&Accuracy=\frac{TP+TN}{TP+FP+FN+TN} \\&MCC=\frac{(TP\times TN)-(FP\times FN)}{\sqrt{(TP+FP)(TP+FN)(TN+FP)(TN+FN)}} \end{aligned}$$

where TP stands for True Positive; FP denotes False Positive; FN denotes False Negative; and TN stands for true negative.

In this study, sensitivity and recall are the same, given that this is a binary classification problem.

### Experimental results and analysis

In this section, we evaluated the performance of DHL-PPI over the above introduced dataset by using recall, precision, *F*1, specificity, accuracy, and MCC score.

The experimental results on the dataset [46] are shown in Table 2. From the table, we can observe that, in general, these results are all very good.

**Table 2:** Performance of DHL-PPI on the the different species in the dataset

Dataset	Recall	Precision	F1	Accuracy	MCC	Specificity
*C. elegans* dataset	0.981	1.000	0.990	0.988	0.975	1.000
Drosophila dataset	0.981	0.998	0.990	0.988	0.976	0.997
*E. coli* dataset	0.962	0.987	0.975	0.971	0.940	0.982
Human dataset	0.963	0.984	0.973	0.971	0.941	0.980

DHL-PPI is tested on the dataset for each of the four species. The data contains the strictly negative examples. In order to compare the other state-of-the-art methods, we only list recall, precision, and *F*1 which were reported by the authors in their own work. Table 3 shows the comparative results with the state-of-the-art methods by Yang et al’s work [43] and Hang et al’s DNN-PPI [37] on the four species.

At first glance of Table 3, one can observe that all the methods produced relatively good results. In these four species, the smallest value is 0.942 which is the recall value of DNN-PPI method in Table  3. Neverthless, the performance of this recall value (0.942) is still very good in general.

The experimental results show that the performance of our proposed DHL-PPI method is competitive with that of Yang et al approach [43], but better than the DNN-PPI method proposed by Hang et al in each of the four species datasets [37]. These results show that our proposed DHL-PPI is suitable to predict PPI in this benchmark data set.

**Table 3:** Performance comparison of different proposed methods on the dataset

Dataset	Method	Recall	Precision	F1
*C. elegans*	DHL-PPI	0.981	1	0.99
*C. elegans*	Yang [43]	0.992	0.993	0.993
*C. elegans*	DNN-PPI [37]	0.981	0.992	0.986
Drosophila	DHL-PPI	0.981	0.998	0.99
Drosophila	Yang	0.996	1	0.998
Drosophila	DNN-PPI	0.9686	0.9995	0.9837
*E. coli*	DHL-PPI	0.962	0.987	0.975
*E. coli*	Yang	0.984	0.994	0.989
*E. coli*	DNN-PPI	0.942	0.975	0.958
Human	DHL-PPI	0.963	0.984	0.973
Human	Yang	0.98	0.995	0.988
Human	DNN-PPI	–	–	–

## Discussion

More effective computational methods for analyzing all-against-all protein–protein interaction (PPI) relationships are needed to reduce the cost and effort of wet-lab experiments. We proposed an effective and feasible method, deep hash learning PPI prediction (DHL-PPI) to predict PPI. Our experimental results suggest that DHL-PPI is not only an effective, but also (at least theoretically) faster than the other state-of-the-arts methods.

On a benchmark dataset with strictly (i.e., experimentally verified) negative and positive PPI examples from four species data, DHL-PPI was shown to be superior to, or competitive with, other state-of-the-art methods.

One might argue that it is hard to draw conclusions based on only one data. However, there are four species inside this dataset, with the smallest having 1528 proteins, while the largest has 7803 samples. Thus, the number of potential all-against-all PPI pairs that may require verification in the four data sets range from $1.166 \times 10^6$ (for *E. coli*) to $3.044 \times 10^6$ (for humans). Furthermore, we mention that this data set is a benchmark data set in the field.

The proposed encoding scheme of DHL-PPI converts the protein sequence into a binary hash code, thus transforming a complicated sequence matching problem into a much simpler and faster problem of finding a number in an array. This process quickly eliminates the irrelevant proteins from further consideration at the pre-screening stage. This suggests that DHL-PPI can serve as a potential encoding scheme to cope with the rapidly increasing volumes of available protein datasets.

The proposed DHL-PPI turns a complex sequence matching problem with time complexity of $O(M^2)$ into a a problem with much lower time complexity $O(M\log M)$. This means that the proposed DHL-PPI is feasible in a database with a large volume of data.

Together, the experimental results suggests that DHL-PPI is feasible and effective in predicting all-against-all PPI relationships in a protein dataset in a faster time.

## Conclusion

In this paper, we proposed a protein–protein interaction prediction model, called DHL-PPI (Deep hash learning protein to protein interaction). DHL-PPI prediction includes an encoding scheme that transforms a protein sequence into a binary hash code and a prediction scheme that predicts potential PPIs using the hash code. Firstly, we encoded a protein sequence into an integer sequence. Secondly, a deep learning technique is applied to generate a better embedding representation for each amino acid. Thirdly, the embedding representation is turned into binary hash codes, namely, ontic hash and query hash codes respectively. At the stage of pre-screening for PPI discrimination, the string matching problem is turned into a much more faster and simpler problem of finding a number inside an array of numbers. For a given query protein, this pre-screening process filters out irrelevant proteins in the dataset that cannot form a PPI with the query protein, resulting a much smaller candidate set for further confirmation. In the final step of PPI discrimination, DHL-PPI uses the Hamming distance between query hash code and ontic hash codes to determine the final PPI relationship set.

We verify the proposed DHL-PPI on a benchmark data set with strictly negative and strictly positive examples from four species. The experimental results confirmed that DHL-PPI is an effective method to predict PPI relationships. Furthermore, at the prediction stage, the proposed DHL-PPI reduce the usual time compexity of $O(M^2)$ to *O*(*MlogM*) for predicting all-against-all pairs of PPI relationships for a database with *M* proteins. DHL-PPI encoding can be applied to a protein dataset with the results stored for later search for potential PPI relationships against a new or unknown query protein.

## Methods

We proposed a protein–protein interaction relationship prediction model, deep hash learning PPI (DHL-PPI), which uses an encoding scheme and a prediction scheme. There are three main stages in the method: preprocessing (“Data preprocessing” section), DHL encoding model (“The DHL encoding model” section), and PPI prediction model and related search algorithms (“PPI prediction model” section). Beside these three steps, in this section, we also explain other important elements of the DHL-PPI model, namely, the loss functions (“Loss functions of the DHL-PPI model” section), and the analysis of the PPI search algorithms (“PPI prediction model” section).

### Data preprocessing

Data preprocessing in DHL-PPI has two key steps. First, each amino acid in a given protein sequence is assigned an interger code by using the Tokenizer API available in Keras [47]. The corresponding code assignments used are shown in Table 4. Then, a sequence consisting of integers is processed by zero-pad method [47] into a sequence with fixed length. This padding step is needed since the neural convolution in the next stage only accepts sequences with fixed length. In this study, the protein sequences with less than 5000 amino acids in the dataset are zero-padded into 5000 amino acids. These sequences are fed into the convolutional neural network (CNN) in the next stage of the DHL model.

**Table 4:** Integer encoding of the amino acids used in the DHL-PPI model

Ala	Gly	VaL	Ile	Leu	Phe	Pro	Tyr	Met	Thr
(A)	(G)	(V)	(I)	(L)	(F)	(P)	(Y)	(M)	(T)
1	2	3	4	5	6	7	8	9	10

Ser	His	Asn	Gln	Trp	Arg	Lys	Asp	Glu E	Cys
(S)	(H)	(N)	(Q)	(W)	(R)	(K)	(D)	(E)	(C)
11	12	13	14	15	16	17	18	19	20

### The DHL encoding model

The DHL-PPI contains two main parts: DHL encoding model and PPI prediction model. The goal of the DHL encoding model is to encode a pair of proteins, say A and B, into a query code and an ontic code, respectively. Then the PPI prediction model uses the encoding to determine whether there is PPI relationship between theses two proteins A and B.

**Fig. 2:**
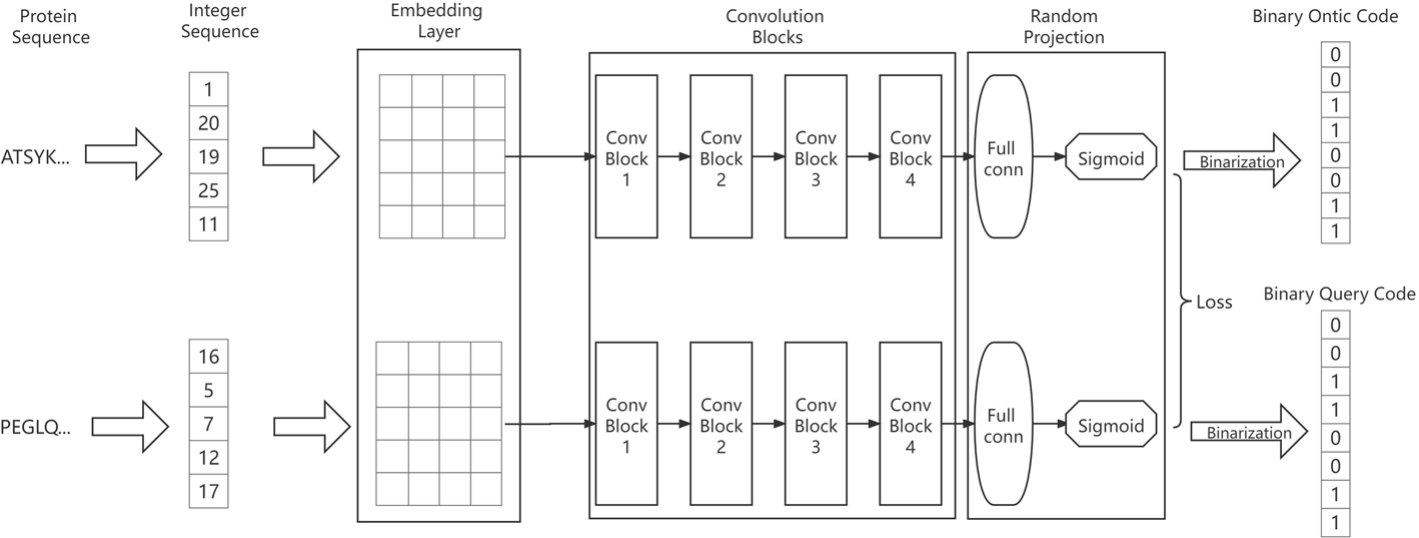
The architecture of DHL-PPI

In turn, the DHL encoding model has four components: the embedding layer, convolution blocks, random projection, and the range control module, as shown in Fig. 2. The proposed DHL model makes an improvement over a basic network proposed in [48]. First, the embedding layer encodes a protein sequence into a vector. Second, the convolution blocks contain four CNN blocks with pairwise inputs. Each CNN contains an embedding layer which tries to generate a better representation for protein sequences. Third, a random projection module is used to transform the vector representations of an ontic sequence and query sequence into an ontic code and a query code, respectively. These two random projections for input proteins A and B are untrainable and they do not share any same parameters. Last, the range control block is implemented as a sigmoid function which limits the output bits into a range of [0,1]. The first two parts and the last part use Siamese networks which share the same network parameters for input proteins A and B.

After these four steps, the protein sequences (A and B) are converted into two respective codes, query binarized hash code and ontic binarized hash code, respectively. Our DHL model is implemented by using Keras API in the Tensorflow2.0 framework.

#### The embedding layer for amino acids

The goal of the embedding layer is to learn a better vector representation of an amino acid, that provides an improved way to encode a protein sequence. This embedding layer maps each protein sequence into a $L*dim$ matrix, where *L* denotes the length of the sequence and *dim* is the number of dimensions for the embedding representation. Each row of the matrix indicates the embedding representation of each amino acid. Before training, *L* is initialized to the length of a protein sequence, and the embedding representation of each amino acid is initialized randomly. In the experiment, *L* is initialized to 5000. In the training process, the embedding layer and convolution blocks are trained together to generate an effective vector to represent a protein sequence. The final value of embedded representation for each amino acid in a given sequence is determined automatically by the model.

#### Convolution blocks

DHL uses four sequential CNN blocks with pairwise inputs. Each CNN contains an embedding layer that tries to generate a better representation for the protein sequence.

Each CNN block operates in four steps. First, it carries out one-dimensional convolutions. Second, it activates the output through ReLU function. Third, it uses batch normalization to reduce the training difficulty. And the final part is pooling. The first three convolution blocks adopt one-dimensional average pooling, while the last convolution block uses global average pooling. The parameters and settings of each block are shown in Table 5.

**Table 5:** The parameters and settings for the convolution blocks in DHL

Block	Number of convolution kernels	Kernel size	Pooling	Stride
ConvBlock1	64	5	1D Average Pooling	1
ConvBlock2	128	7	1D Average Pooling	1
ConvBlock3	256	9	1D AveragePooling	1
ConvBlock4	512	15	1D Global Average Pooling	1

#### Random projection module

The DHL model contains two different random projection modules. These two random projections are untrainable and they do not share parameters. One random projection module transforms a sequence into an ontic code. The other random projection module transforms the same sequence into a query code. Each random projection module contains a sub-network of fully connected layer with 64 neurons. These two random projection modules accept the same input from the previous convolution module. And then this same input are mapped into an ontic code and a query code, respectively. The ontic and query codes are respective 64-dimensional vectors. In order to do this, the weights of these two random projection modules are set to be different and untrainable. These untrainable characteristics reduce the number of trained parameters and further speed up the training process to avoid the risk of over-fitting.

#### Range control module

The range control module, which is the last part of DHL, uses a sigmoid function on the 64-dimensional vector representations from the previous step. The sigmoid function is used to force the output into the range of [0,1]. It combines with the hash constraint loss to minimize the quantization loss $x-sign(x)$. The final output is binarized using the sign function (Eq. 1), that is, the sigmoid is used to approximate the sign function.

1 $$\begin{aligned} sign(x)={\left\{ \begin{array}{ll} 1&{} \text { if x }\ge 0.5\\ 0&{} \text {otherwise} \end{array}\right. } \end{aligned}$$

### Loss functions of the DHL-PPI model

The DHL-PPI model has three types of losses, namely, discrimination error loss ($\mathcal {L}_d$), hash constraint loss ($\mathcal {L}_h$), and bit balance loss ($\mathcal {L}_b$).

Discrimination error loss occurs when using the query hash code to search against candidate ontic hash codes to determine whether there is interaction relationship between two proteins. Hash constraint loss occurs during the stage of binarization of hash codes. Bit balance loss occurs during the stage of optimizing the generalized hash codes to obtain a balance between 0 and 1 distribution.

#### Discrimination error loss

If there is an interaction between two proteins, the distance between their corresponding hash codes should be closer, otherwise, the distance should be larger. Specifically, when training the DHL-PPI model, if two proteins have a known interaction, the distance threshold $in_{dist}$ between them is set to 2 or smaller (in the range of [0, 2] ). If two proteins do not have any known interaction (or strictly no interaction is confirmed), the distance threshold $unin_{dist}$ should be greater or equal to 12. The definition of the discrimination error loss function is shown in Eq. 2:

2 $$\begin{aligned}&\mathcal {L}_d =label\times max(dist-in_{dist},0)+(1-label)\times max(12-unin_{dist},0) \end{aligned}$$

3 $$\begin{aligned}&dist=\sum _{i=1}^N|HC^{ont}_i-HC^{que}_i| \end{aligned}$$

where *N* represents the dimension of the hash code, $HC^X_i$ is the *i*-th bit position in the hash code representation of sequence *X*, and *label* is the label of a protein pair. If there is interaction between two proteins, then $label=1$ , otherwise $label=0$. The $in_{dist}$ and $unin_{dist}$ represent the respective minimum and the maximum distance between protein hash codes, which is set to 2 and 12, respectively. Equation 3 shows a way to calculate the distance, *dist* used in Eq. 2. In Eq. 3, the *dist* is the Manhattan distance between hash codes from two proteins, query code and ontic code. When each bit in the hash code is close to 0 or 1, *dist* will be close to the Hamming distance. The hash bit values $HC^{ont}_i$ and $HC^{que}_i$ are the *i*-th bit of the ontic code corresponding to protein *A* and the query code corresponding to protein *B* before binarization, respectively. In this study, *N* is set to 64, thus each hash code is just a simple integer on a 64-bit machine.

#### Hash constraint loss

Hash constraint loss occurs in the stage of binarization of hash codes. In some related study [49–51], the tahn (tangent) function is used to binarize the hash code in training phase. See also [52] for other approaches to hash code generation. The proposed DHL-PPI uses the sigmoid function to limit the model output to [0,1] first. And then it introduces the hash constraint loss shown in Eq. 4 to minimize the constraint loss. Specifically, it makes each hash code as close as possible to 0 or 1 before binarization. In this way, the total hash code loss function is minimized.

4 $$\begin{aligned} {\mathcal{L}}_{h} & = {\mathcal{L}}_{h}^{{ont}} + {\mathcal{L}}_{h}^{{que}} \\ {\mathcal{L}}_{h}^{{ont}} & = max(quan_{{thresh}}^{2} \times N - \sum\limits_{{i = 1}}^{N} {(HC_{i}^{{ont}} - quan_{{thresh}} )^{2} } ,0) \\ {\mathcal{L}}_{h}^{{que}} & = max(quan_{{thresh}}^{2} \times N - \sum\limits_{{i = 1}}^{N} {(HC_{i}^{{que}} - quan_{{thresh}} )^{2} } ,0) \\ \end{aligned}$$

where $\mathcal {L}_h^{ont}$ and $\mathcal {L}_h^{que}$ denote the hash constraint loss of ontic code and query code, respectively. The total hash constraint loss $\mathcal {L}_h$ is the sum of these two quantities. $quan_{thresh}$ is the quantization threshold, which is set to 0.5 in this case. When each bit of the hash code is close to 0 or 1, the loss is smaller. Otherwise, when each bit is 0.5, the loss will be the largest.

#### Bit balance loss

When encoding a protein sequence into a binary hash code in DHL-PPI, the ideal situation is that the probability for each bit to be 0 or 1 should be the same. However, in practice, this ideal is not always the case. The bit balance loss is used to capture the difference between the ideal and real situation. The bit balance loss function is defined in Eq. 5.

5 $$\begin{aligned} {\mathcal{L}}_{b} & = {\mathcal{L}}_{b}^{{ont}} + {\mathcal{L}}_{b}^{{que}} \\ {\mathcal{L}}_{b}^{{ont}} & = (mean(HC^{{ont}} ) - 0.5)^{2} \\ {\mathcal{L}}_{b}^{{que}} & = (mean(HC^{{que}} ) - 0.5)^{2} \\ \end{aligned}$$

where $\mathcal {L}_b^{ont}$ and $\mathcal {L}_b^{que}$ represent the bit balance loss of ontic code and query code respectively; $HC^{ont}$ and $HC^{que}$ represent a binary hash ontic code and a binary hash query code respectively. The total bit balance loss is the sum of these two values. When the average of the hash code is $\frac{0+1}{2}=0.5$, the bit balance loss is the minimum. Accordingly, when the generated binary hash codes are balanced (that is, the possibility of binarized hash code being 1 or 0 is the same), the total bit loss is minimal. Otherwise, when the average is far away from 0.5, the loss will be the largest.

#### Total loss Function

The total loss is the sum of the above three mentioned loss functions, namely, the discrimination loss, the hash constraint loss and the bit balance loss, which is shown in Equation 6. This loss function is incorporated into DHL-PPI to train the model iteratively to optimize the final output, binarized hash codes.

6 $$\begin{aligned} \mathcal {L} =\lambda _d\mathcal {L}_d + \lambda _h\mathcal {L}_h +\lambda _b\mathcal {L}_b \end{aligned}$$

where $\mathcal {L}_d$ is the discrimination error loss, $\mathcal {L}_h$ is the hash constraint loss and $\mathcal {L}_b$ is the bit balance loss; and $\lambda _d, \lambda _h$, and $\lambda _b$, are corresponding weights for each type of loss.

The sigmoid function is given as $f(z)=\frac{1}{1+exp(-z)}$. Its derivative is $f'(z)=f(z)(1-f(z))$. When *f*(*z*) is close to 0 or 1, its derivative tends to 0. The design objective in DHL-PPI is to minimize the total error during training. Ideally, the output of *f*(*z*) should be close to 0 or 1. This would lead to the disappearance of the gradient. Therefore, in this study, we set the weights as follows: $\lambda _d=\lambda _h=1$, and $\lambda _b=2/N$, where *N* is the dimension of the hash code, set at $N=64$ in this study. In the earlier stage of the training, the discrimination loss and bit balance loss play more important roles in model fitting. In the later training stage, with the decreasing of the discrimination loss, the hash constraint loss comes into play and constraints the hash code to 0 or 1.

### PPI prediction model

Here, we describe our approach to PPI prediction (sometimes also referred as PPI discrimination or PPI identification). The state-of-the-art deep learning methods usually contain two parts, one is encoding and the other is discriminating. The proposed DHL-PPI only utilizes the encoding part and does not use the traditional discrimination stage in the deep learning model. That is, the deep learning techniques in DHL-PPI are only used to generate the required binary hash codes (query code and ontic code) for the given protein sequence. In the prediction phase, DHL-PPI first conducts a pre-screening process to narrow down the whole database. And then it compares a query code to an ontic code by calculating the Hamming distance between them.

In the pre-screening stage, the proposed DHL-PPI searches a binarized hash query code against all binarized hash ontic codes in a database. Because they are all binarized codes, these digital bits can be regarded as a binary number. The problem of searching a protein sequence against all proteins (i.e, *M* number of protein sequences) in a database is turned into a problem of finding a binary number (an integer) inside an array of size *M*. This pre-screening process significantly narrows down the subset of the database that need to be further analyzed to verify the hypothesised PPIs.

In the final step of PPI prediction, DHL-PPI will compare the binarized hash query code against an ontic code using the Hamming distance. This Hamming distance measurement is used as criteria to determine whether there is a relationship between two proteins. This is another advantage of the proposed DHL-PPI which is faster and simpler than the other methods. Generally, computing the Hamming distance is much faster than using a traditional discriminator in deep learning model.

Let us examine the Hamming distance comparison and the threshold used in this study. In the information theory, the Hamming distance between two equal-length strings is the number of different symbols at corresponding positions of the strings. That is, the number of symbols needed to be replaced by converting one string to another string. In this step, an important parameter is the distance threshold, *d*, which determines whether there is interaction between two proteins or not. Specifically, if the Hamming distance is greater than *d*, it is assumed that there is no interaction between these two proteins, otherwise, there is. In this study, we set the distance threshold $d=4$.

Given a protein sequence *P* and a protein database *D* which contain *M* number of proteins. The problem is to determine whether the given *P* has any interaction (relationship) with any other protein inside the database *D*. The intuitive way is to compare a given protein sequence *P* with all proteins inside the database *D* one by one which requires *M* number of comparisons (the number of proteins inside *D*). If one wishes to determine whether there is any interaction between every pair of proteins in the database *D*, (the all-against-all PPI problem), we need to perform $M(M-1)$ sequence comparisons, since each of the *M* proteins in the database *D* will need to be compared against the other $M-1$ proteins.

In the pre-screening stage of PPI prediction, we convert the sequence comparison problem into a much simpler problem of finding a number in an array. Next, a binary query code of a protein *P* is used to search on the binary ontic codes by calculating the Hamming distance. Using the proposed DHL-PPI prediction method, the comparison time is reduced significantly. In the proposed DHL-PPI prediction method, this can be done in $O(M\log _2M)$ comparison operations. The detailed implementation is explained in the next subsection.

#### The PPI prediction algorithms

Here, we introduce the PPI prediction algorithms. Algorithm  1 is the overall process of PPI prediction. There are three input parameters in the algorithm. One is the Hamming distance threshold, *d*, which determines whether there is a relationship between two proteins. The second one is the ontic codes of a protein database, *D*, which are generated in the previous DHL model. The third is the query code of a protein database, *Q*, which is also generated in the previous DHL model. The output parameter *PPIset* contains the predicted PPI relationships.

Let’s examine the algorithm line by line. In Line 1, set *PPIset* is initialized to empty. And *M* is assigned to the number of proteins in the query codes of database *Q*. In Line 2, the indexes of ontic codes of database *D* are built. There are total of $C_{2d}^{d}$ different indexes given a known Hamming distance threshold, *d*. Please see more detailed information in the following indexing step. The for loop from Line 3 to Line 7 checks each protein’s query code in *Q*. In Line 4, it takes a query code $q=Q[i]$ from database *Q*. In Line 5, it calls the binary code searching algorithm (BCSA) to obtain the predicted results *T*. In Line 6, the relationship of query *q* and the predicted results *T* is added into the output set *PPIset*. After all members inside database *Q* are checked throughout, the final result is returned in Line 8.
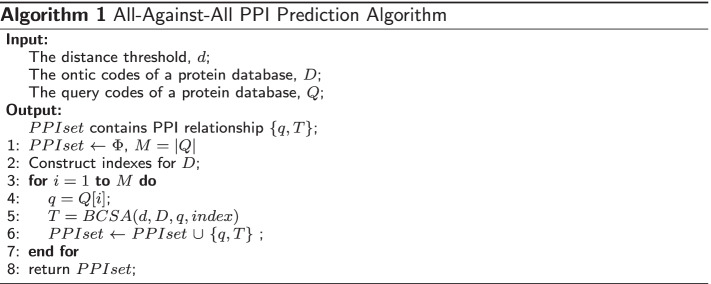


Two key steps in Algorithm  1 require further explanation. One is in Line 2, the process to build indexes for an ontic codes of database *D*. The other is the binary code searching algorithm (BCSA) called in Line 5. Both of these are discussed in the following subsection. Before that, we first introduce the Pigeonhole principle, a key concept used in this work.

#### The Pigeonhole principle

Given two sequences each with length of *N*, if the Hamming distance between them is less than or equal to *d*, after dividing these two sequences into 2*d* fragments evenly, inside these 2*d* fragments (hence, each fragment will be of size *N*/2*d*), there are at most *d* number of fragments which are different, that is, the remaining *d* fragments would have the same exact sequences.

The Pigeohole principle is utilized in the pre-screening step. According to the Pigeonhole principle, in 2*d* fragments, if one finds that *d* fragments are the same, then we can claim that the distance between these compared sequences is less than or equal to *d*. To choose *d* parts from 2*d*, there are $C_{2d}^{d}$ combinations. In this study, we have $d=4$. Then, the possible number of combinations in choosing *d* from 2*d* fragments will be: $C_{2d}^{d}=70$.

In the pre-screening step, the algorithm searches all these $C_{2d}^{d}$ situations to identify possible protein candidates whose distance are less than or equal to *d*. For example, in this study, if the distance between two sequences is less than or equal to 4, then at least four out of the eight parts have the same exact bit patterns, that is, at least half of the 64 bits (32 bits) in the hash codes are the same. These 32 bits can be regarded as numbers. In this way, we can turn the sequence comparison problem to a much simpler problem of searching a number inside an array. This operation greatly improves the pre-screening process and narrows down the candidate set.

#### The indexing process

The goal of indexing process is to build indexes which are binary numbers, and then turn sequence comparison problem into a much simpler problem of finding a number inside an array.

The indexing process are conducted as follows. First, the protein binarized hash codes are divided into 2*d* fragments. And then, *d* fragments are randomly selected from these 2*d* fragments. The exact same *d* fragments are chosen from all ontic codes in a database. Finally, these *d* fragments are connected together serving as an index code for a protein.

Remembering that, the ontic binary hash codes and query binary hash codes are binary numbers, an index by concatenating random *d* fragments from 2*d* fragments together is still a binary number. There are a total of $C_{2d}^{d}$ indexes.

For a database containing *M* proteins, it takes $O(M \log _{2} M )$ time to build all indexes and sort an array with length *M* in order. Totally, the time requirement is $O(C_{2d}^{d} M \log _{2}M)$. When $d=4$ (the distance threshold value used in this work), $C_{2d}^{d}=70$, this value can be regarded as a constant value. Thus, the time complexity of index building is $O(M \log _{2} M )$.

#### BCSA: binary hash code searching algorithm

The binary hash code searching algorithm (BCSA) is a nearest-neighbor filtering algorithm based on the binarized bash code. This is shown in Algorithm 2. There are four input parameters in the algorithm: (1) the distance threshold, *d*, where $d=4$ in this work; (2) The ontic hash codes of protein database, called *D* here; (3) the corresponding index of ontic hash codes, called *idx*, which is constructed during the indexing process; (4) a query hash code of the queried protein, called *q*.
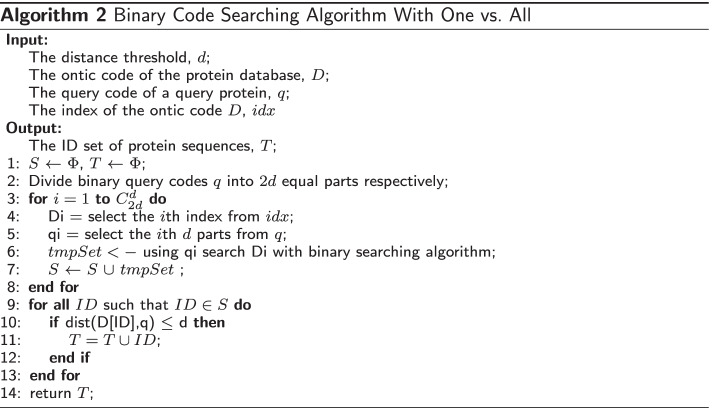


In Algorithm 2, in Line 1, the candidate set *S* is set to empty, the output PPI relationship set *T* is also set to empty. In Line 2, the query hash codes *q* are divided into 2*d* fragments evenly. From Line 3 to Line 8, there is a for loop which goes through each possible combinations ($C_{2d}^{d}$) in the query codes *q*. Inside the for loop, in Line 4, the corresponding *i*th-index inside *D* is chosen, called $D_i$. Note that, $D_i$ is an array containing $M=|D|$ number of binary integers here. That is, $D_i$ is an sorted array with *M* numbers inside. The same *d* fragments from binary hash query code are chosen from query protein, called $q_i$, which is one binary number (Line 5). In line 6, it compares the number $q_i$ against sorted array $D_i$ by using binary search algorithm. That is, it tries to find all numbers inside array $D_i$ which are equal to $q_i$. In this way, the string matching problem of searching a sequence against a database is turned into a simpler problem of finding an exact number in an array. Thus, all proteins whose indexes which have the exact value as $q_i$ are saved into Candidate Set *S* (Line 7).

When all indexes are processed, the Candidate Set *S* contains all proteins who at least have one of their *Di* equal to *qi*. Note that the number of proteins in Set *S* is much less than the number of proteins in the original dataset *D*. From Line 9 to Line 13, the algorithm further uses Hamming distance to compare the query code *q* to each ontic code whose protein ID is in the Candidate Set *S*. The protein who has the distance which is less than and equal to the threshold *d* ($d=4$ in this study) is selected as a potential PPI and placed in set *T*.

#### Analysis of the algorithms

We examine the time complexity of the PPI prediction algorithms here.

Let’s first check Algorithm 2. In Line 1, the Candidate Set *S* and *T* is set to empty, which done in *O*(1) constant time. In Line 2, dividing a query code *q* into 2*d* fragments evenly requires time $O(C_{2d}^{d})$. Taking *d* parts out of 2*d* fragments, there are $O(C_{2d}^{d})$ possible combinations. From Line 3 to Line 8, the for loop checks each possible combination one by one. In Line 4 and Line 5, it takes *d* parts out of 2*d* parts from ontic codes *D* and the query code *q* respectively. The time complexity for Line 4 and Line 5 are *O*(1). In Line 6, it uses the binary hash code for *qi* (a number) to search against a sorted array $D_i$ by using the binary search algorithm. The time complexity for Line 6 is $O(\log _{2} M)$, where *M* is the number of protein sequences inside database *D*. Thus, using binary search, all the sequences in the database *D* whose $D_i$ are equal to $q_i$ are saved into the Candidate set *S* (Line 7). Let the number of search results be denoted $K_{i}$. The time complexity for Line 7 is $O(K_{i})$. Together, the time complexity of the for loop from Line 3 to Line 8 is: $O( C_{2d}^{d}(\log _{2}M +\sum {K}_{i}))$. From Line 9 to Line 13, all sequences inside Candidate Set *S* is verified by using Hamming distance. Pairs of sequences with distances less than or equal to the threshold *d* are then added to the final output set *T*. The time complexity for Line 9 to Line 13 is $O(\sum {K}_{i})$.

Together, the time complexity for Algorithm 2 is $O(C_{2d}^{d}(\log _{2}M + \sum _{i=1}^{C_{2d}^{d}}{K_{i}}) + \sum _{i=1}^{C_{2d}^{d}}{K_{i}}) = O(C_{2d}^{d}(\log _{2}M + \sum _{i=1}^{C_{2d}^{d}}{K_{i}}))$. For example, in this study, $d=4$ and $C_{2d}^{d}=70$, both of them can be regarded as constant, then the time complexity is $O(\log _{2}M + \sum _{i=1}^{C_{2d}^{d}}{K_{i}})$.

Let’s consider the average situation for $\sum _{i=1}^{C_{2d}^{d}}{K_{i}}$. Here, $K_i$ is the number of exact matches found during each search inside the for loop. With *M* proteins in the database *D*, $K_i$ is in the range of [0, *M*]. The ontic code and the query code are binary sequences each of length 64. Then, in each index search, there are $\frac{d}{2d} \times 64=32$ bits to be checked. On average, the possibility of exact match is $\frac{1}{2^{32}}$. For a database *D* with *M* proteins, since the hash codes are generally balanced, the probability of an exact match is : $\frac{M}{2^{32}}$. The expected value for one loop (one search) is : $O(\log _{2}M + \sum _{i=1}^{C_{2d}^{d}}{\frac{M}{2^{32}}})$. In this study, M < 40,000 (this covers the number of human genes). For simplicity, let’s assume M is equal to a larger value, say 40,000 here. Then, we have $\sum _{i=1}^{C_{2d}^{d}}{\frac{M}{2^{32}}}=C_{2d}^{d} \frac{M}{2^{32}}=\frac{70*40{,}000}{2^{32}}<\frac{2,800,000}{10^9}<<1$. Thus, this value can be regarded to be 1. Then, the time complexity for the above formula $O(\log _{2}M + \sum _{i=1}^{C_{2d}^{d}}{\frac{M}{2^{32}}})$ can be rewritten to be: $O(\log _{2}M +1) = O(\log _{2} M )$. This is the time complexity for Algorithm 2.

Now, let’s consider the time complexity of Algorithm 1. Line 1 runs in O(1). In Line 2, the time complexity of constructing indexes for database *D* is $O(C_{2d}^{d}M\log _{2}M)$. From Line 3 to Line 7, the loop traverses all the query codes of the protein database *Q* which has *M* protein sequences. Both Line 4 and Line 6 run in O(1). Line 5 calls Algorithm 2 which runs in $O(\log _{2} M )$. The time complexity of the for loop is $O(M\log _2M)$. Then, the total time complexity of Algorithm 1 is $O(C_{2d}^{d}M\log _{2}M+M*\log _2M)$. In this study, the distance threshold *d* is set to 4 and $C_{2d}^{d}=70$, and both of them are constant. Thus, the time complexity is $O(M log_2 M )$. That is, we can claim that the PPI prediction algorithm runs in $O(M log_2 M )$ time complexity, where *M* is the number of proteins inside a database. This is a big improvement over the state-of-the-art $O(M^2)$.

## Data Availability

Our source codes and data can be found in URL:https://github.com/labSAlin/DPPI. The Protein–Protein Interactions (PPI) dataset is from the article, entitled, “Large-scale prediction of human protein–protein interactions from amino acid sequence based on latent topic features” [46]. The dataset is available at the author’s website, URL:http://www.csbio.sjtu.edu.cn/bioinf/LR_PPI/Data.htm.
